# Quantifying Changes in Vaccine Coverage in Mainstream Media as a Result of the COVID-19 Outbreak: Text Mining Study

**DOI:** 10.2196/35121

**Published:** 2022-09-20

**Authors:** Bente Christensen, Daniel Laydon, Tadeusz Chelkowski, Dariusz Jemielniak, Michaela Vollmer, Samir Bhatt, Konrad Krawczyk

**Affiliations:** 1 Department of Mathematics and Computer Science University of Southern Denmark Odense Denmark; 2 Department of Infectious Disease Epidemiology MRC Centre for Global Infectious Disease Analysis Imperial College London London United Kingdom; 3 Department of Management in the Network Society Kozminski University Warsaw Poland; 4 Section of Epidemiology Department of Public Health University of Copenhagen Copenhagen Denmark

**Keywords:** data mining, COVID-19, vaccine, text mining, change, coverage, communication, media, social media, news, outbreak, acceptance, hesitancy, understanding, knowledge, sentiment

## Abstract

**Background:**

Achieving herd immunity through vaccination depends upon the public’s acceptance, which in turn relies on their understanding of its risks and benefits. The fundamental objective of public health messaging on vaccines is therefore the clear communication of often complex information and, increasingly, the countering of misinformation. The primary outlet shaping public understanding is mainstream online news media, where coverage of COVID-19 vaccines was widespread.

**Objective:**

We used text-mining analysis on the front pages of mainstream online news to quantify the volume and sentiment polarization of vaccine coverage.

**Methods:**

We analyzed 28 million articles from 172 major news sources across 11 countries between July 2015 and April 2021. We employed keyword-based frequency analysis to estimate the proportion of overall articles devoted to vaccines. We performed topic detection using BERTopic and named entity recognition to identify the leading subjects and actors mentioned in the context of vaccines. We used the Vader Python module to perform sentiment polarization quantification of all collated English-language articles.

**Results:**

The proportion of front-page articles mentioning vaccines increased from 0.1% to 4% with the outbreak of COVID-19. The number of negatively polarized articles increased from 6698 in 2015-2019 to 28,552 in 2020-2021. However, overall vaccine coverage before the COVID-19 pandemic was slightly negatively polarized (57% negative), whereas coverage during the pandemic was positively polarized (38% negative).

**Conclusions:**

Throughout the pandemic, vaccines have risen from a marginal to a widely discussed topic on the front pages of major news outlets. Mainstream online media has been positively polarized toward vaccines, compared with mainly negative prepandemic vaccine news. However, the pandemic was accompanied by an order-of-magnitude increase in vaccine news that, due to low prepandemic frequency, may contribute to a perceived negative sentiment. These results highlight important interactions between the volume of news and overall polarization. To the best of our knowledge, our work is the first systematic text mining study of front-page vaccine news headlines in the context of COVID-19.

## Introduction

Theoretical models suggest that the herd immunity threshold for SARS-CoV-2 requires at least two-thirds of the population to be immunized through either natural infection or vaccination [1]. Though multiple safe and effective vaccines have been developed [2-4], one significant challenge in achieving pandemic control is “vaccine hesitancy,” which ranges from mistrust to outright refusal of vaccination [5].

Vaccine hesitancy extends beyond COVID-19 and is 1 of the 10 biggest threats to global health according to the World Health Organization (WHO). At its core, vaccine hesitancy is an issue of perception, rooted in the information individuals receive [6].

Social media is an important source of both vaccine information and misinformation. Although vaccine-related tweets are predominantly positively polarized [7], there is also substantial (possibly coordinated) misinformation [8] that contributes to vaccine hesitancy [9]. Further, the volume of tweeted fake news within a given country negatively correlates with its vaccine uptake [10]. Antivaccination supporters on Twitter share more conspiracy theories and make greater use of emotional language than provaccination supporters [11]. Moreover, vaccine discourse is highly politicized [12], and the likelihood of endorsing misinformation is ideologically driven [13,14].

Different sides of vaccine discourse prioritize different objective values: Arguments in favor of vaccines prioritize community, while arguments against vaccines focus on individual freedom [15]. A high proportion of parents' opinions on vaccines expressed online is aggressive, accusatory, or inaccurate [16].

Major news outlets also play an important role in vaccine discourse [17,18]. Although several text mining studies have covered vaccines within specific regions [19-22], to the best of our knowledge, there are no large-scale text mining studies to date of vaccine front-page news headlines that encompass multiple countries focusing specifically on COVID-19.

Here, we analyzed online news media coverage of COVID-19 vaccines. We used text mining analysis to estimate the volume of online vaccine news coverage during 3 time periods: (1) before the COVID-19 pandemic, (2) before the COVID-19 vaccine announcement, and (3) after the COVID-19 vaccine announcement. We used ~28 million front-page headlines collected from 11 different countries with a healthy online news media ecosystem, defined using SimilarWeb traffic and BBC media profiles [23]. Because sentiment toward vaccines is influenced by the context in which they are mentioned, the most frequently mentioned topics were gathered alongside the most frequently mentioned companies and organizations. Our analysis aimed to inform future public health and vaccine communication, with a view to hopefully reducing vaccine hesitancy.

## Methods

### Curation of a Front-page News Article Database

We analyzed the landing pages from major online news sources (ONSs) in countries with a healthy media ecosystem. The data are fully described in a previous study [23] that focused on front-page news from 172 leading ONSs in 11 countries (Australia, Canada, France, Germany, Ireland, Italy, New Zealand, Russia, Spain, the United Kingdom, the United States) and an international category. The international category contained headlines from ONSs that were internationally distributed (eg, EuroNews or AlJazeera). The data used articles published from July 2015 to April 2021, which covered the following 3 time periods: (1) before the outbreak of COVID-19, (2) during the pandemic before the COVID-19 vaccine announcement, and (3) during the pandemic after the COVID-19 vaccine announcement. We took November 2020 as the cutoff date for the COVID-19 vaccine announcement, as from this point on, the press started covering SARS-CoV-2 vaccines following the announcement by BioNTech and Pfizer. We note this date applies to western countries, which are the subject of our study, and is less applicable globally. The updated data set included a total of 28,709,060 headlines, from which 14,638,278 were in the English language and 14,070,782 were in a language other than English.

### Identifying Vaccine Headlines

Keywords were used to identify whether a given headline was vaccine-related. For non-English headlines, keywords were supplied by native speakers. For English headlines, we supplied the keywords ourselves. The keywords used can be found in Table 1.

Non-English headlines were stemmed using SnowballStemmer [24] and case-folded (Table 1) to capture the equivalence class of different forms of words (eg, the German words Impfung, impfen, Impfgegner all map to impf). English headlines were lemmatized using TreeTagger [25], all words were case-folded, and punctuation was removed, whereby words connected by a hyphen were separated into 2 words. English headlines were lemmatized to avoid misclassifications (eg “immunity” understood in a legal rather than a biomedical sense).

The techniques used to identify vaccine headlines varied by language, and we used the same methodology as in our previous work [23]. In French, Italian, Russian, and Spanish, titles and descriptions were tokenized, and if either the title or the description contained at least one keyword, the headline was labeled as a vaccine headline. In English and German, titles and descriptions were kept as strings, and a search was performed for keyword patterns. If a keyword pattern was present, the headline was designated as a vaccine headline (eg, in German, the prefix Impf-). Machine learning translation offers an alternative way to identify vaccine headlines across languages; however, this was beyond the scope of this work.

**Table 1:** Keywords used to identify the vaccine headlines.

Language	Keywords
English	vaccin immunis immuniz anti vax antivax
French	vaccin antivaccin immunis
German	impf
Italian	vaccin antivaccin immunizz
Russian	прививк привива вакцин иммунизац вакцинац
Spanish	vacun antivacun inmuniz

### Splitting the Data Into 3 Vaccination-Specific Periods

We divided the data into 3 time periods: (1) the pre-COVID-19 era, (2) during the pandemic before the COVID-19 vaccine announcement, and (3) during the pandemic after the COVID-19 vaccine announcement. This division of the data was based on clear changes within media coverage with respect to vaccines and COVID-19. On January 9, 2020, daily media coverage of the coronavirus began, so we chose this date as the end of the pre-COVID-19 era. We chose November 9, 2020, as the cut-off date separating the prevaccine and after-vaccine announcements. This resulted in the following 3 periods:

1. Before COVID-19: July 2015 to January 8, 2020
2. Before the COVID-19 vaccine announcement: January 9, 2020, to November 9, 2020
3. After the COVID-19 vaccine announcement: November 10, 2020, to April 2, 2021

To identify changes in each period, the relative frequency of vaccines mentioned in the full data set, along with the relative frequency of headlines containing either “COVID-19” or “coronavirus,” was calculated at weekly intervals using equation 1.



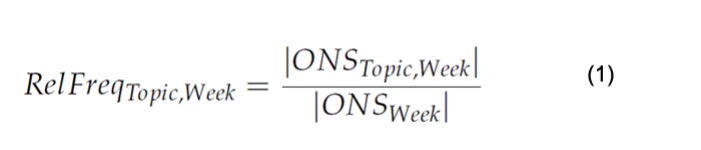



where |*ONS_Topic,Week_*| is the number of headlines on a particular topic in a given week and |*ONS_Week_*| is the number of headlines in that same given week. The relative frequency was calculated first with respect to vaccines, where all vaccine-related headlines were included, and second with respect to COVID-19, where all headlines containing either the keyword “coronavirus” or “COVID-19” were included.

### Topic Detection of the Vaccine Headlines in the 3 Periods Using BERTopic

Topics were identified for 91 English ONSs using BERTopic. Topics were not identified for the non-English ONSs, as finding the optimal number of topics within non-English ONSs would require languages to be handled separately and would also require in-depth knowledge about each language. BERTopic is a topic modelling technique that uses a combination of transformers and c-TF-IDF to create dense clusters using HDBSCAN, where c-TF-IDF is a class-based TF-IDF that can be used to generate features from text [26]. We chose to use BERTopic as it was previously successful in heterogeneous text mining [27,28] and it offers multiple pretrained models. Additionally, scatterplots of the embeddings of the data from the 3 periods did not show a clear clustering of the headlines, which rules out several other topic detection techniques (please see Figures S1-S3 in Multimedia Appendix 1).

To remove patterns from the text input to BERTopic that could otherwise affect the model, all abbreviations, links, and names referring to the different newspapers were removed. Additionally, the word “news” was removed, along with words containing “immuniz,” “immunis,” and “vaccin,” which were used to extract the vaccine headlines. The phrases “anti vax” and “antivax” were retained, as they refer to resistance toward vaccination.

Text input to BERTopic was normalized to reduce word variation. The headlines were lemmatized using TreeTagger combined with case-folding. TreeTagger is a tool for annotating text with part-of-speech and lemma information using a Markow tagger, which uses a decision tree to obtain reliable estimates. TreeTagger was also used to remove filler words from headlines by only using words tagged as either a noun (including proper nouns), verb, or adjective and removing words that contained little information about topics.

**Figure 1:**
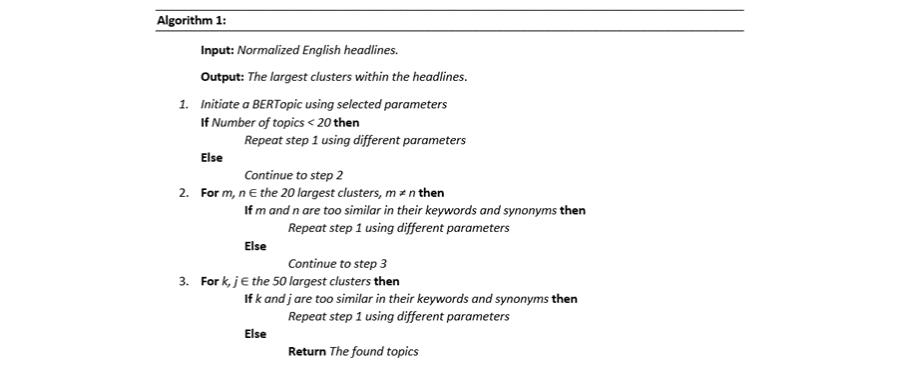
Pseudocode for the 2-step evaluation method to identify the number of clusters reflecting the most common topics.

We employed a 2-step evaluation method to identify the number of clusters reflecting the most common topics (Section 1 in Multimedia Appendix 1). The pseudocode for this is illustrated in Figure 1. Evaluating topic similarity (step 2) was performed manually, as 2 topics might deal with the same subject but contain several seemingly different keywords or word combinations, which would make the model split them into 2 topics instead of 1 topic. Therefore, the decision of how to continue from step 2 was likewise done manually.

### Named Entity Recognition of Vaccine Headlines Using SpaCy

Named entity recognition (NER) identifies and categorizes words (or strings of words) for an entity, where an entity can be the name of a person, organization, location, or work of art. We used NER to determine the companies and organizations that were mentioned frequently in the context of vaccination. NER was performed on both English and non-English data using SpaCy with different pipelines depending on the language. SpaCy is an advanced natural language processing tool that is able to perform NER on multiple different languages using statistical models. Therefore, it uses previous training and predictions to decide whether a word or collection of words is a named entity and which kind of entity it most likely is [29]. Pipelines were chosen according to the reported accuracy by SpaCy. In all cases, the most accurate pipeline was used, which were en_core_web_trf, de_core_news_lg, fr_core_news_lg, it_core_news_lg, ru_core_news_lg, and es_core_news_lg. The 2 first letters in each pipeline refer to the language for which it was trained.

Entities such as “AstraZeneca-Oxford” or “Pfizer-BioNTech” were split to count as separate entities. The occurrences of “Johnson and Johnson” and “J&J” were altered to “Johnson & Johnson.”

Individual entities were enumerated using case-folded entities. We created 2 bar plots (see Multimedia Appendix 1), one containing the 30 most frequently occurring named entities from English ONSs and another containing the 30 most frequently named entities from non-English ONSs.

### Frequent N-grams With Respect to the Different Vaccine Manufacturers

Changes in sentiment toward vaccination before and after the COVID-19 vaccine announcement were determined by assessing 7 frequently occurring vaccine manufacturers found using NER. A data set containing English headlines for each vaccine manufacturer was created, which was then assessed with respect to frequent bigrams and trigrams (referred to as n-grams henceforth). The lemmatized headlines created for the topic detection were used for this purpose.

For all vaccines and periods, the 50 most frequent n-grams were assessed. In some cases, a combination of 2 bigrams, with almost the same count as a trigram, would combine to give that trigram. For instance, the bigrams (*food, drug*) and (*drug, administr*) combined give the trigram (*food drug administr*). This was caused by “Food and Drug Administration” in some cases being referred to as “Food and Drug Authority” or “Food and Drug Association.” Such bigrams were removed, keeping only the trigrams. Similar bigrams were excluded for “Food and Drug Administration,” “Centers for Disease Control,” and “European Medicines Agency.” Additionally, “FDA,” “CDC,” “NIH,” “WHO,” and “EMA” were commonly occurring abbreviations among the frequent words with respect to some vaccines, which were added to the number of occurrences of “Food and Drug Administration,” “Center for Disease Control,” “National Institute of Health,” “World Health Organization,” and “European Medicines Authority,” respectively. Other abbreviations such as “NHS,” “HHS,” and “PHE” were assessed with respect to frequent bigrams and trigrams. Likewise, if bigrams occurred the same number of times as a trigram containing the bigram, the bigram was removed.

### Sentiment Analysis of the Vaccine Headlines of 3 Periods Using VADER

We performed sentiment analysis on English-language headlines using VADER [30]. Before assessing sentiment values, each headline’s raw score was calculated using the positive and negative sentiment values in equation 2:

*Raw_score_*=*Positive_score_* - *Negative_score_*
**(2)**

The extent of negative or positive sentiment polarization varied between ONSs and over time. Therefore, a comparison of sentiment toward vaccines between the periods and ONSs on the raw sentiment values would not show whether a change in sentiment toward vaccines was due to an overall change in sentiment or, instead, due to a change in sentiment specifically toward vaccines. Therefore, to enable comparison of the periods and between the ONSs, each sentiment value for a vaccine headline was adjusted according to the overall average sentiment in the given ONS. The adjustment was done using the VADER sentiment values (either raw or compound, denoted by *S_ONS,Topic,Period_*), subtracting the mean sentiment value for the same ONS, with respect to nonvaccine headlines in the same period (either raw or compound, denoted by 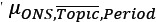).

This is referred to as the relative sentiment skew (RSS) and is given in equation 3:



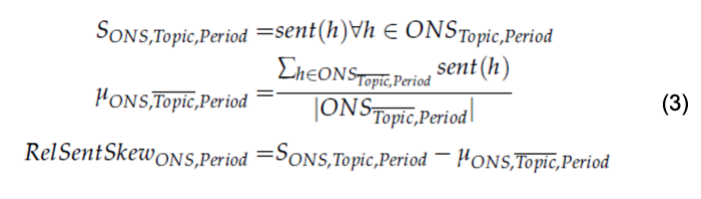



where *ONS_Topic,Period_* is the collection of headlines of a given topic for a given ONS in a specific period, 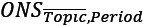 is the collection of headlines not pertaining to that topic for that same ONS in all periods, *h* is a single headline, and *sent(h)* is the sentiment value of h, while 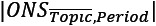 is the number of headlines not in the given topic for that same ONS in all periods. In this case, the topic in equation 3 is vaccines. The raw scores were used to RSS each headline, with respect to the 3 periods. These were illustrated in line plots, in which the cumulative frequency showed the proportion of negative and positive RSS values of a certain smaller value. Because of the nuanced nature of the news, we applied the same manual checks here as in our previous work to make sure sentiment annotations were correct [23].

## Results

Of the 14,638,278 English-language headlines identified over all 3 data periods, 83,395 (0.6%) were found to be vaccine-related using the keywords defined in Table 1. Dividing these with respect to the 3 periods gave the following number of vaccine headlines within each period: (1) before COVID-19: 11,361; (2) before the COVID-19 vaccine announcement: 17,112; (3) after the COVID-19 vaccine announcement: 54,922.

### Large Increase in Ratio of Vaccine Headlines With the Rollout of COVID-19 Vaccines

**Figure 2:**
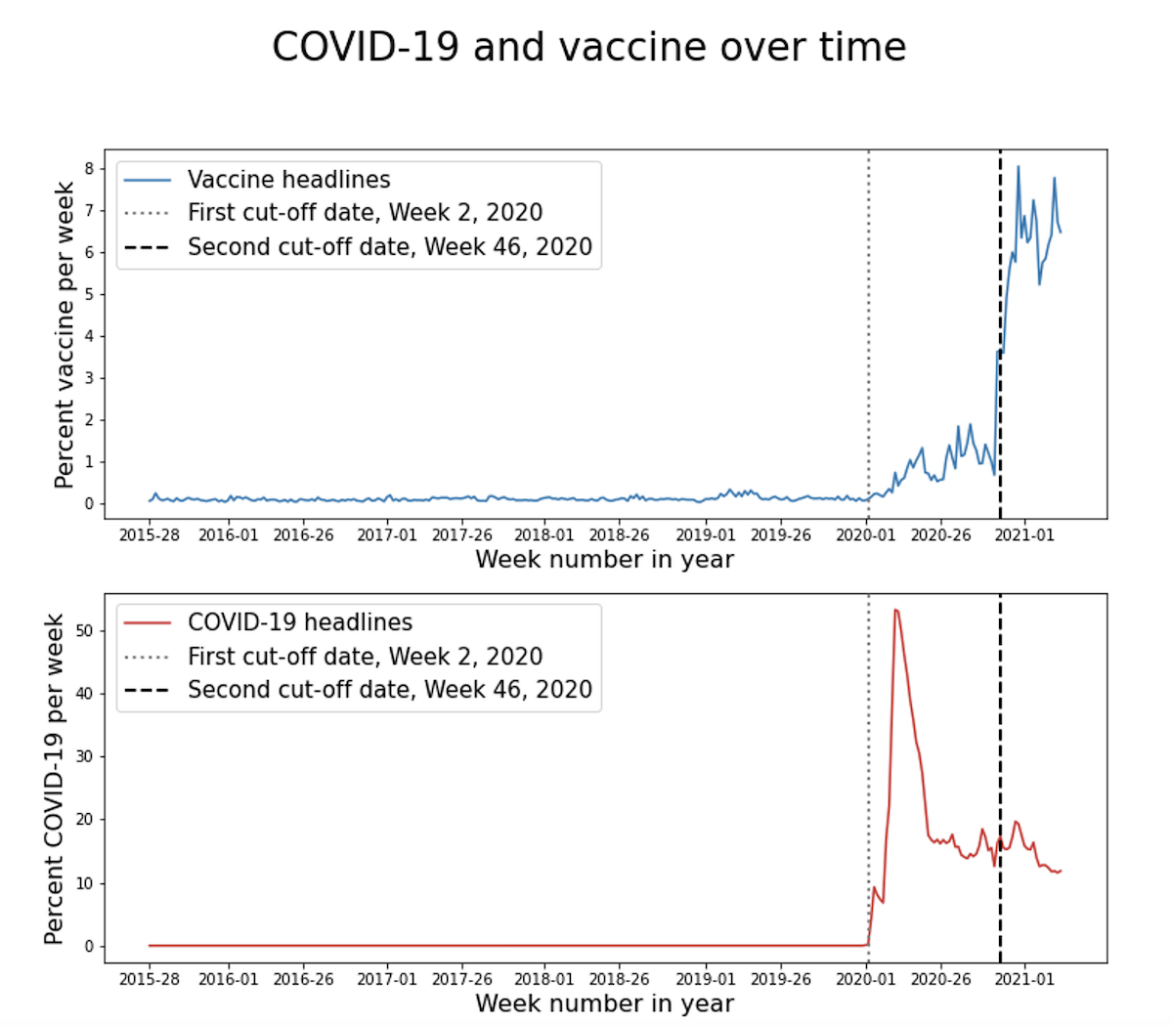
Percentage of headlines mentioning (A) vaccines and (B) "COVID-19" or "coronavirus" in the mainstream media over time, with the first and second cut-off dates (dotted and dashed vertical lines, respectively).

We calculated the percentage of vaccine coverage within newspaper headlines for each week within each time period of data collection, plotted in Figure 2. Before the pandemic, the percentage of vaccine headlines was low (0.1% across 172 ONSs). With the COVID-19 outbreak in early 2020, the proportion of vaccine headlines increased to an average of 4%.

**Figure 3:**
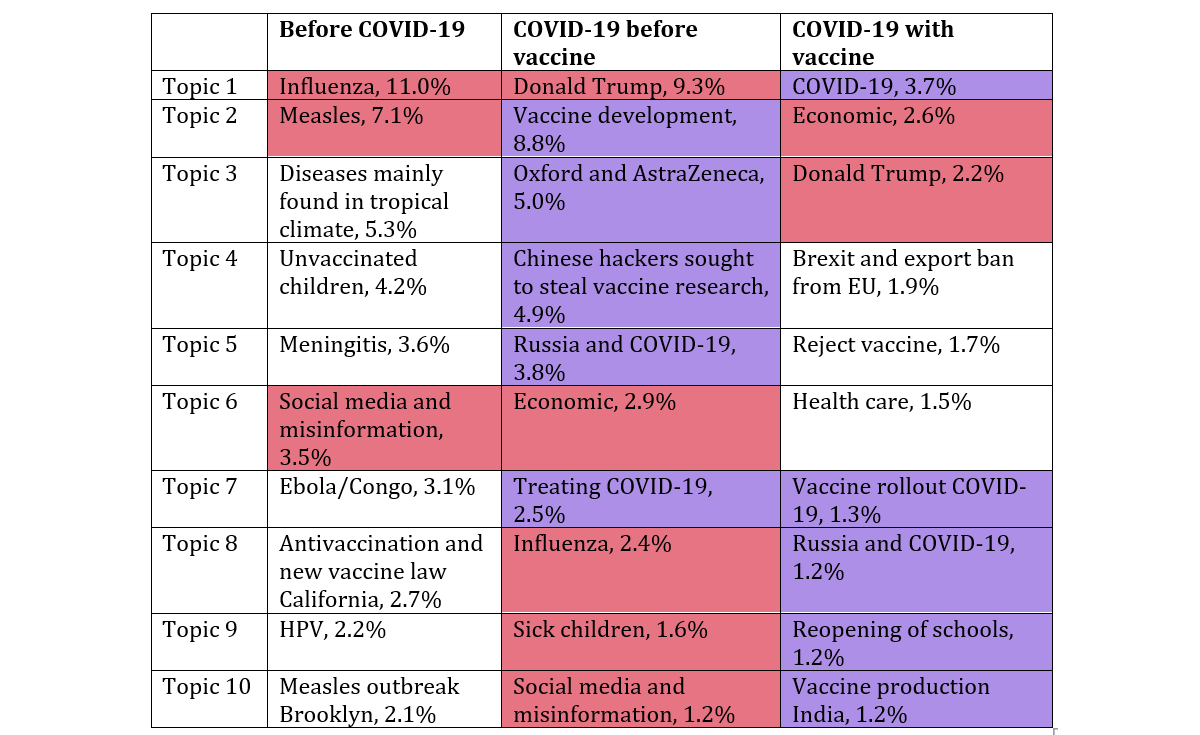
The 10 most common topics within vaccine-related articles during the 3 time periods. Purple cells highlight topics directly related to COVID-19, while red cells highlight topics that occur during more than one period. Notice that “Russia and COVID-19” is colored purple even though it occurs in multiple periods. EU: European Union; HPV: human papillomavirus.

Increased reporting on vaccines during the second period coincided with the advent of COVID-19 reporting. The 10 most common topics in vaccine coverage in the 3 periods are shown in Figure 3. Causal connections cannot be established, as the COVID-19 coverage reached one-quarter of all front-page coverage with nuanced associations with reported topics [23]. Unsurprisingly, the most common vaccine-related topics during the second and third time periods were related to the pandemic. Although COVID-19 increased vaccine news coverage, coverage of COVID-19 was not directly correlated with that of vaccine coverage (Figure 2).

Rather than dropping to a stable level, as COVID-19 headlines did (Figure 2), the proportion of vaccine headlines increased from week 45 to week 47 of 2020 to between 6% and 8% and remained at this level until April 2, 2021. This increase is linked to the Pfizer and BioNTech press release on November 9, 2020, which reported 90% effectiveness in preventing COVID-19, paving the way for the rollout in the United Kingdom beginning on December 2, 2020.

**Figure 4:**
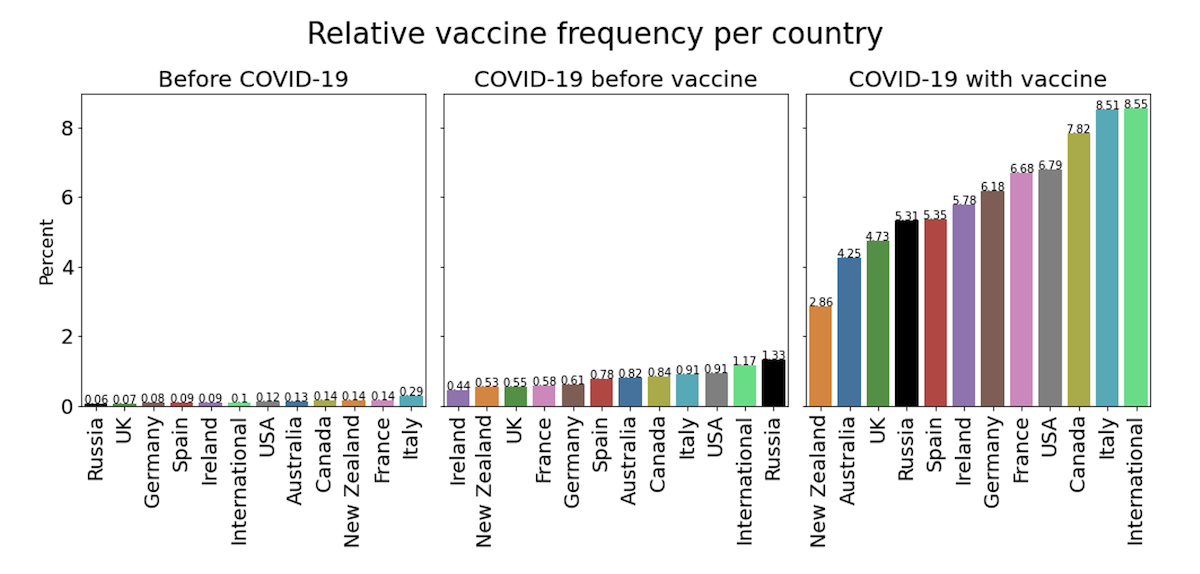
Relative vaccine frequency for each country including the international online news sources for each of the 3 periods: (A) before COVID-19, (B) before the COVID-19 vaccine announcement, and (C) after the COVID-19 vaccine announcement.

Relative frequencies of vaccine headlines were calculated for each period and each country (Figure 4). Relative frequencies for each country were similar, with very limited attention toward vaccines before the pandemic and a steep rise after the introduction of the first SARS-CoV-2 vaccine.

### Majority of Vaccine Reporting Had Positive Sentiment Polarization With the Outbreak of COVID-19 as Opposed to the Prepandemic Era

**Figure 5:**
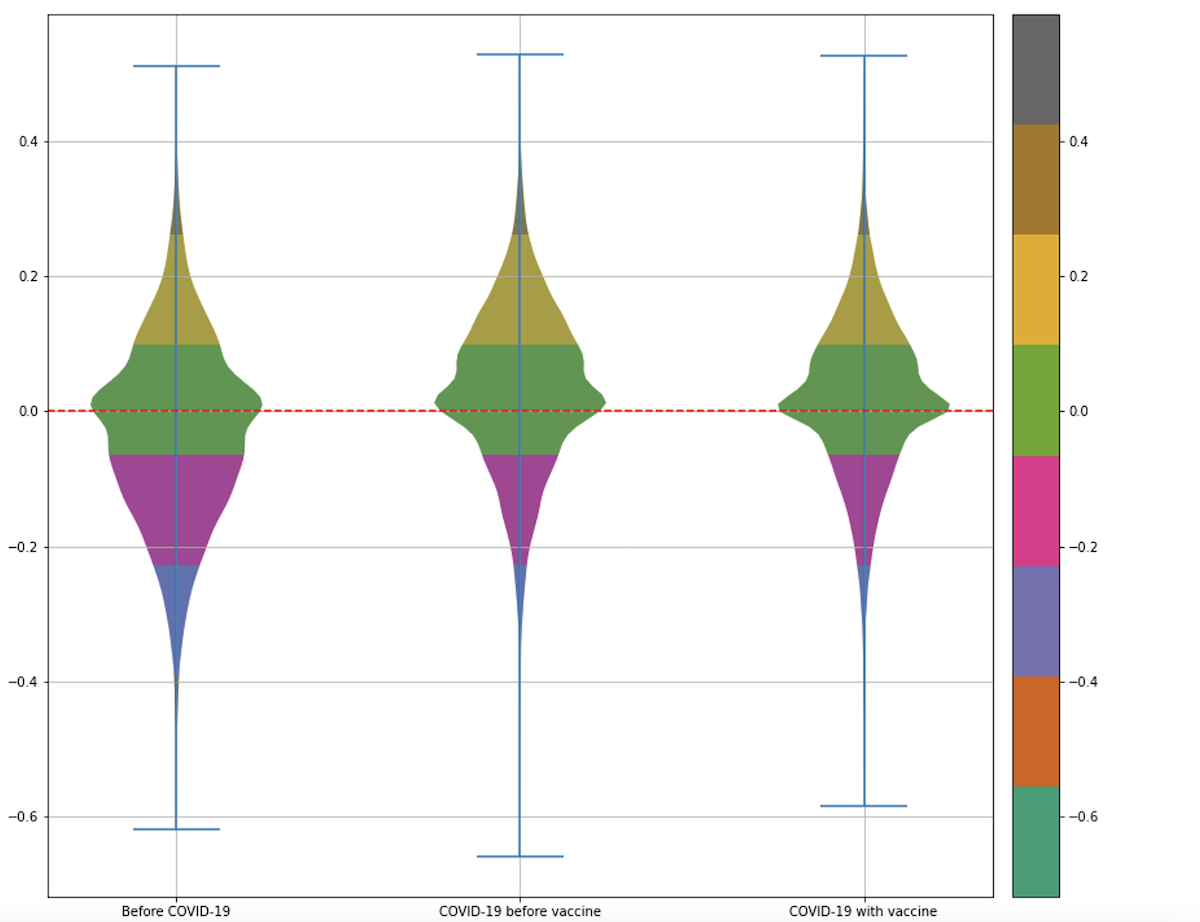
Relative sentiment skew (y axis) of vaccine coverage in the 3 periods used in this study.

Figure 5 shows the VADER sentiment scores for vaccine-associated headlines within each time period. The increased frequency of vaccine reporting during the pandemic led to an increase in the absolute number of negatively polarized articles, from 6698 in 2015-2019 to 28,552 in 2020-2021. Overall, however, polarization during the pandemic was majority positive (38% negatively polarized) as opposed to the prepandemic period, when 57% of articles were negatively polarized. Figure 3 suggests that the difference in sentiment between pre-COVID-19 and post-COVID-19 vaccine coverage could be associated with COVID-19 coverage. This could be because COVID-19 became the dominant topic globally, accounting for one-quarter of all news during the pandemic.

To investigate the difference in sentiment distribution between the 2 periods during the pandemic, we contrasted the topics and named entities mentioned in both periods. The period “Before the COVID-19 vaccine announcement” can largely be interpreted as the period in which all vaccines were under development, while “After the COVID-19 vaccine announcement” is the period in which some vaccines were rolled out and others were still under development. Although there is a difference between the periods before COVID-19 and after COVID-19, there was not a sizable sentiment discrepancy between the 2 periods during the pandemic (Figure 5).

We further investigated the topic polarization of the articles relating to the COVID-19 vaccine development and rollout. We extracted articles associated with 2 topics from Figure 3: “Vaccine development” and “Vaccine rollout.” One could argue that “Vaccine production” (topic 10) should be merged with “Vaccine rollout” in line with our interpretation of the periods. However, we wanted to avoid manual intervention in topic annotations. The individual articles were extracted from the data giving 2 data sets of approximately the same size (846 and 814 headlines, respectively).

**Figure 6:**
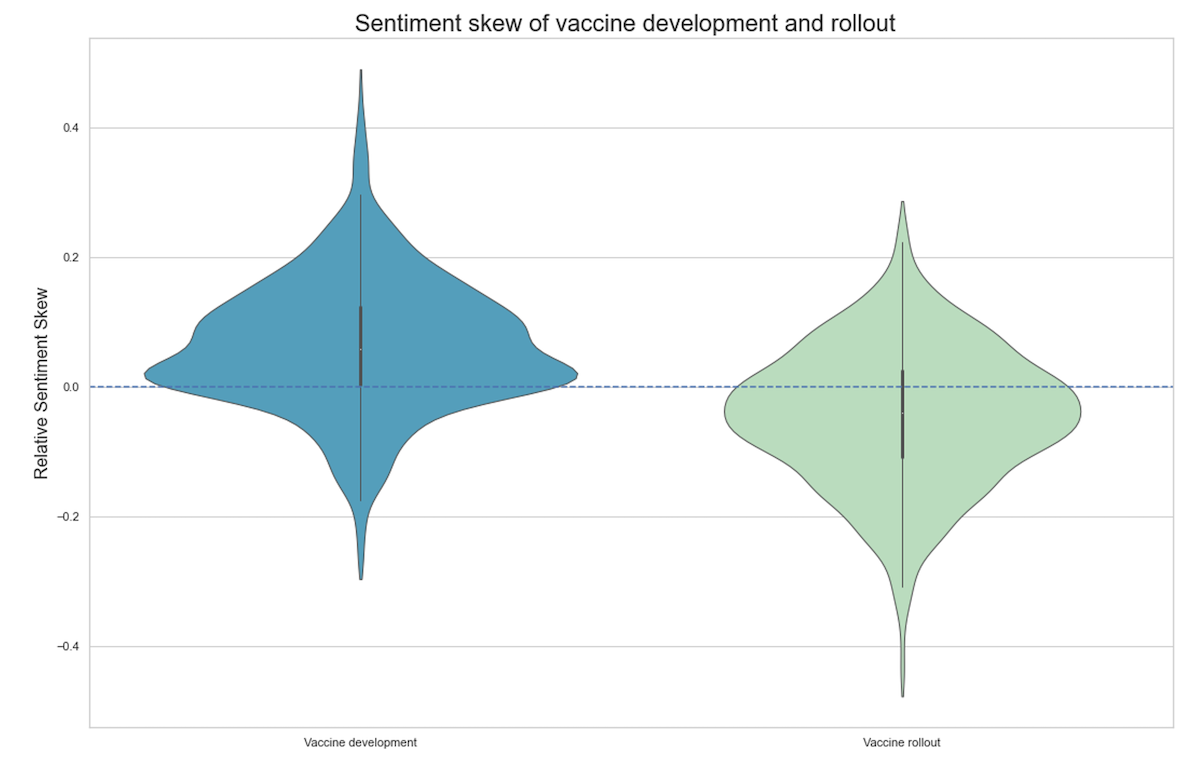
Relative sentiment skew for the topics "Vaccine development" and "Vaccine rollout" using the raw sentiment value.

We assessed sentiment polarization of the topics “Vaccine development” and “Vaccine rollout.” RSS of raw VADER sentiment for “Vaccine development” and “Vaccine rollout” is illustrated in Figure 6, which shows a change in vaccine sentiment between the development and trial phase and the rollout of the vaccines. Figure 6 illustrates that, for “Vaccine development,” sentiment is overwhelmingly positive, with almost the entire interquartile range above the zero line. Of the headlines in “Vaccine development,” 23% had negative RSS, while 77% had positive RSS. This is very different from “Vaccine rollout,” for which 66% had negative RSS and only 34% had positive RSS. Additionally, the widest area lies above zero for “Vaccine development” and below zero for “Vaccine rollout.” Therefore, the RSS with the highest frequency is positive for “Vaccine development” and negative for “Vaccine rollout.” The largest and smallest RSS for the 2 topics are quite different: “Vaccine Development” lies in the range from –0.3 to just below 0.5, while “Vaccine rollout” lies in the range from –0.5 to 0.3; so, their RSS values are equally spread, but their ranges are differently situated. This suggests that the difference in sentiment distributions between the 2 COVID-19 periods could be attributed to more negative coverage during vaccine rollout.

### Most Common Organizations Mentioned in the Context of COVID-19 Vaccines and Sentiment Toward Them

**Figure 7:**
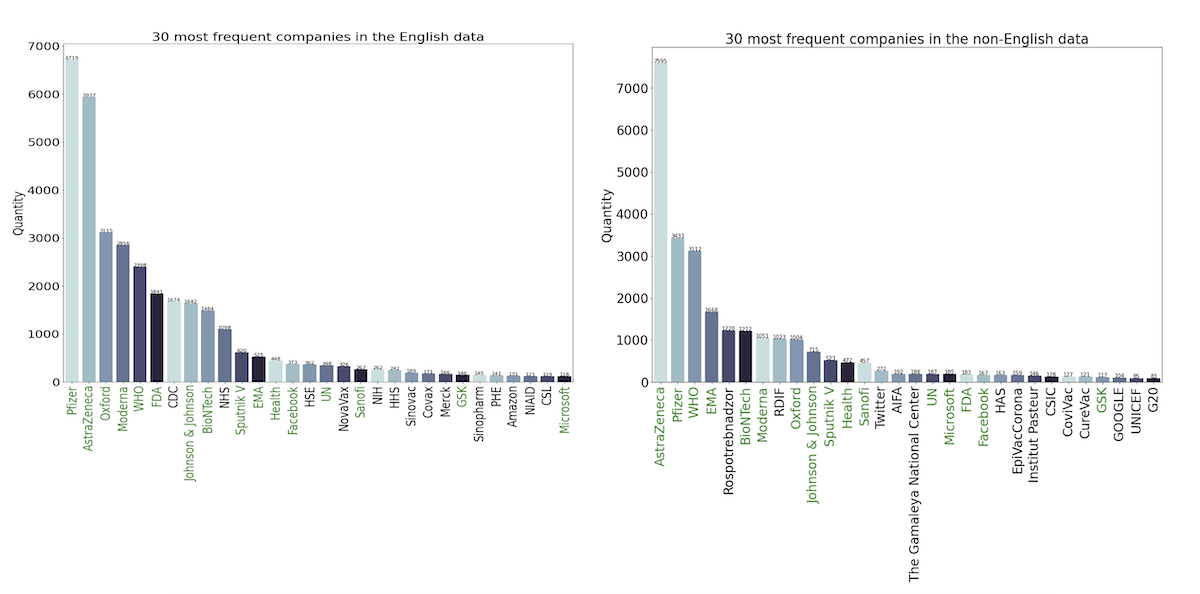
The 30 most frequent entities (companies and organizations) found in the (A) English and (B) non-English data. The green names are the organizations and companies that were found in both English and non-English data.

To gain more granular insight into sentiment polarization during the pandemic period, we investigated the top entities mentioned. We employed SpaCy to perform NER, and the 30 most frequently mentioned companies or organizations for all 3 periods are illustrated in Figure 7.

Unsurprisingly, the most common associations were between well-known COVID-19 vaccine manufacturers, namely “AstraZeneca” (in collaboration with Oxford), “Pfizer” (in collaboration with BioNTech), “BioNTech,” “Moderna,” “Oxford,”, “Johnson & Johnson,” and “Sputnik V.” Though AstraZeneca and Oxford, as well as Pfizer and BioNTech, developed their vaccines as a partnership, they were frequently mentioned separately; thus, we opted to keep them as separate entities.

Of the 30 most frequent named entities, in both English and non-English headlines, 16 occurred in both data sets, colored green in Figure 7. The nonoverlapping entities were mainly attributed to national organizations or companies. For instance, “NHS” and “HHS” are the National Health Service and the Department of Health and Human Services from the United Kingdom and United States, respectively, and were solely found among the 30 most frequent English entities. “Rospotrebnadzor” is the Federal Service for Surveillance on Consumer Rights in Russia, and “RDIF” and “PAH” are also Russian and were found solely among the 30 most frequent non-English entities. Additionally, company names are the same across different languages, whereas some national organizations are not; for instance, the abbreviation for the World Health Organization is WHO in English, while in French, it is OMS.

The frequency at which vaccine manufacturers were mentioned within all news headlines increased from almost zero before COVID-19 to most frequently mentioned within the period after the vaccine announcement (Table 2). Therefore, vaccine manufacturers were assessed only within the COVID-19 pandemic.

The most common associations with vaccine manufacturers indicated progress in development and rollout and were health-related (eg, side effects). Detailed analysis of the n-grams for each vaccine developer are in Section 2 of Multimedia Appendix 1. Vaccines by Moderna and Pfizer were chiefly associated with n-grams indicating progress of clinical trials and their rollouts. By contrast, top n-grams associated with AstraZeneca and Johnson & Johnson were linked to side effect reporting (eg, unexplained illness, blood clot). Throughout the pandemic, Sputnik V was mentioned not in a medical context but rather frequently linked to Russia and Vladimir Putin, containing frequent n-grams like *“*Soviet Union,” “President Vladimir Putin,” and “Russia Soviet Union.”

**Figure 8:**
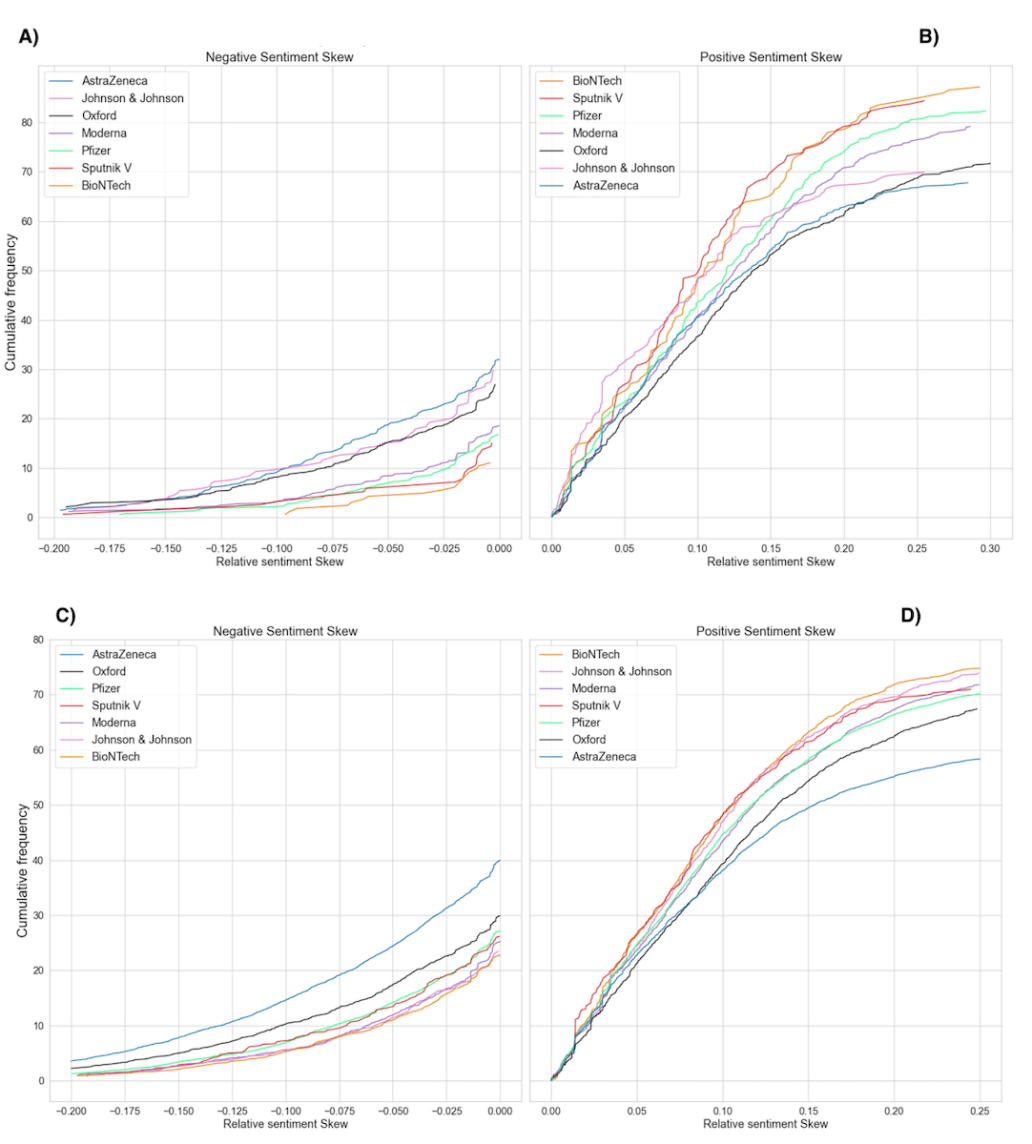
Proportion of negative and positive sentiment polarization with respect to entities associated with vaccine manufacturing in the periods “Before the COVID-19 vaccine announcement” and “After the COVID-19 vaccine announcement”: (A) negative sentiment skew "Before the COVID-19 vaccine announcement," (B) positive sentiment skew "Before the COVID-19 vaccine announcement," (C) negative sentiment skew "After the COVID-19 vaccine announcement," (D) positive sentiment skew "Before the COVID-19 vaccine announcement".

We investigated the extent to which the difference in the context of vaccine manufacturers influenced news article sentiment. In Figure 8, we plotted the proportion of negative and positive sentiments toward the vaccine manufacturer entities before and after the vaccine announcement. In the period before the COVID-19 vaccine announcement, entities appear to have similar negative polarizations, AstraZeneca and Johnson & Johnson being noted as slight outliers with more negative coverage. After the COVID-19 vaccine announcement, AstraZeneca had a notably higher ratio of negative articles and a lower ratio of positive articles. Despite Johnson & Johnson being associated with side effects (as per our n-gram analysis), AstraZeneca received notably worse press. We removed AstraZeneca coverage from Figure 5 and Figure 6 to test whether the higher associated volume of negative news influenced the slightly more negative polarization in the phase after the COVID-19 vaccine announcement. In both cases, we did not find that AstraZeneca was the main driver in more negatively polarized articles in that period (please see Tables S1 and S2 in Multimedia Appendix 1).

**Table 2:** The 21 different subsets created with respect to the different vaccines and periods, including the number of times each of the different manufacturers were mentioned within the news headlines in each subset (7 vaccine manufacturers in 3 periods).

Manufacturer	Before COVID-19	Before the COVID-19 vaccine announcement	After the COVID-19 vaccine announcement
AstraZeneca	3	747	5134
BioNTech	1	163	2118
Johnson & Johnson	17	332	1050
Moderna	3	647	2256
Oxford	3	1010	2288
Pfizer	27	513	6042
Sputnik V	0	153	700

## Discussion

We used text mining to study vaccine reporting on the front pages of top national news outlets. We demonstrated that reporting on vaccines increased in volume from coverage of around 0.1% on front pages to almost 4% of all headlines during the pandemic. Despite reporting covering the vaccines’ side effects, overall coverage can be classified as positive, in line with previous studies of social media that reported positive polarization of vaccine-related tweets [7].

The news ecosystem accounts for 76% of the information people consume [31], which can affect people’s behavior, for instance making them more hesitant toward vaccines. This can be exacerbated by circulation of misinformation [21] and by vaccine reporting along partisan lines [19].

However, news is only one facet of the entire media ecosystem, and much information is communicated via social media [19-22]. Social media encourages active participation in the form of clicks, likes, retweets, and shares, which are then readily quantifiable by engagement. With news however, the engagement is much more nuanced, especially because of more passive information consumption when people merely scan headlines. Nonetheless, traditional news is still vital in forming opinions and, in many cases, constitutes the initial discourse on other platforms.

We focused on analyzing headlines from a handful of western countries to provide a data-centric analysis of vaccine coverage across several countries. Similar studies have been conducted in individual countries (eg, Brazil [22]) or other regions (eg, Africa [20]). Our study encompasses countries that were among the first to manufacture and introduce the vaccine on a large scale (United States, Russia, Germany, United Kingdom). In these countries, policy makers had to navigate vaccine hesitancy and ongoing COVID-19 restrictions with sophisticated media coverage throughout the development and rollout phases.

We analyzed how front-page headline vaccine reporting evolved during the COVID-19 pandemic. For the analysis, we made a set of assumptions that are associated with certain limitations. Our focus on the headlines in predominantly developed western countries underrepresents the situation faced in other parts of the world that were also affected by COVID-19, where vaccine hesitancy is compounded by inequality in vaccine manufacturing and distribution [32,33]. We justify using headline information by virtue of normalizing heterogeneous long-form texts across different news sites and by capturing the behavior of passive scanning of headlines. However, this introduces a disconnect between the information in the full article that might not be reflected in an attention-attracting headline and thus leads to different information consumption by the reader. Within our data set, we opted for a keyword-based approach that was previously used to measure the extent of COVID-19 reporting [23]. The approach is designed to increase the precision of identified headlines, though at the expense of recall. For instance, the headline “UK measles outbreak: 500,000 British children don’t have crucial jab - Daily Star. MORE than half a million children in the UK didn’t receive a…” was not extracted for the English vaccine data set, as it does not contain any of the chosen key words given in Table 1, even though it clearly pertains to vaccination. Developing a more complex topic model would not guarantee better performance and comparability between different languages, as one would have to develop a suitable model that captures the same linguistic nuances. Therefore, we resorted to simple mentions of basic vaccine-derived keywords to aid comparison across countries.

Even though this approach underestimates the number of vaccine-related articles, COVID-19 vaccine reporting was still given central prominence, unlike before the outbreak when vaccines were covered only sporadically. Studying the volume of vaccine coverage motivated our division of the data into the 3 periods, before COVID-19, during COVID-19 but before the vaccines, and with COVID-19 vaccines. It is possible that our definitions of the second and third periods could have influenced our results. However, we found it reasonable to make these divisions according to the large rise in the relative frequency in vaccine headlines due to the Pfizer and BioNTech press release on November 9, 2020. This press release influenced all countries, while many of the other cornerstones in this period were more country-specific. For instance, the United Kingdom was the first country to approve the Pfizer-BioNTech vaccine on December 2, 2020, with the United States Food and Drug Administration approval of the Pfizer-BioNTech vaccine occurring on December 11, 2020.

Our topic modelling and sentiment analysis showed that COVID-19 increased the proportion of vaccine headlines by more than an order of magnitude, from a negligible 0.1% to a formidable 4% during vaccine rollout across 172 ONSs. Reporting on vaccines prior to COVID-19 was negatively polarized. By contrast, vaccine-related reporting during the pandemic is positively polarized. Though we note a discrepancy in sentiment polarization pre- and post-COVID-19, this could be attributed to sampling bias post-COVID-19, as there was significantly more vaccine coverage. Moreover, sentiment polarization in the headlines might not relate directly to vaccines but rather to tangential topics. We therefore also analyzed the tendences in sentiments relating to specific concepts or entities, such as vaccine development or vaccine manufacturers.

We performed in-depth sentiment analysis of the subtopic AstraZeneca, which received more negative coverage because of widely reported side effects and delivery issues. According to our analysis, however, such negative reporting was not significant enough to alter the overall positive narrative of vaccines in the news. Although The University of Oxford co-created the vaccine, it does not experience an equally large proportion of negative headlines as does AstraZeneca, which might be reflected in the media coverage frequency of the 2 with respect to vaccines. Although AstraZeneca is mentioned 5881 times during the pandemic, Oxford is mentioned 3298 times, mostly in the period before the COVID-19 vaccine announcement, while for AstraZeneca the majority is in the subsequent period. Therefore, AstraZeneca is more frequently connected with the vaccine in the media coverage than Oxford.

Our findings study the online news media’s vaccine coverage and are also applicable more widely to general mistrust of authority and science. Although direct connections between news coverage and vaccine uptake are beyond the scope of this study, we have comprehensively characterized sentiment toward COVID-19 vaccination in the online news media. Future survey-based studies into vaccine hesitancy will hopefully benefit from our work, as it details the changing information landscape on which the public ultimately base their decisions. Our work is therefore also important for public health policy makers who require knowledge of the information that the public consumes when designing vaccine mandates.

## Appendix

Multimedia Appendix 1 Supplementary information.

## Abbreviations

NER: named entity recognition
NIHR: National Institute for Health Research
ONS: online news source
RSS: relative sentiment skew
VEEPED: Vaccine Efficacy Evaluation for Priority Emerging Diseases
WHO: World Health Organization
